# Biodefense: Research Methodology and Animal Models

**DOI:** 10.3201/eid1303.061488

**Published:** 2007-03

**Authors:** Kim Taylor

**Affiliations:** * National Institutes of Health, Bethesda, Maryland, USA

**Keywords:** biodefense, research, countermeasures, book report

Since the terrorist attacks of September 11, 2001, biodefense research has been increasingly emphasized. Biodefense: Research Methodology and Animal Models, edited by James R. Swearengen, is a timely and invaluable reference for those performing animal experimentation to develop medical countermeasures and diagnostics against infectious agents and toxins identified as potential biological weapons. Those persons not involved in this field of study who want to educate themselves on biodefense research will also find it interesting and informative. It clearly and concisely provides extensive details about the animal models, both past and present, that have been used to investigate a selected number of disease processes caused by exposure to plausible biological threat agents.

The book is exceptionally well written and furnishes a wealth of information from world-renowned scientists who spearheaded infectious disease research at the United States Army Research Institute of Infectious Diseases. This reference will equip researchers with pertinent information regarding current animal models, so that future work will not repeat experiments already performed, while at the same time minimizing the number of animals projected for future biodefense studies. Most chapters are devoted to a specific biological agent or toxin and supply interesting historical information as well as descriptions about the pathogenesis in humans and in animal models. Specifically, this reference discusses in detail the bacterial agents that cause anthrax, glanders, plague, tularemia, and Q fever; the viral agents that result in Venezuelan and Eastern and Western encephalitis, poxviruses and hemorrhagic fever viruses; and finally, toxin- and superantigen-induced diseases caused by botulinum toxins, ricin, and staphylococcal and streptococcal bacteria. An entire chapter is devoted to the challenges associated with aerobiology. The information in this chapter is well organized and completely outlines the topic, to include transporting and caring for aerosol-challenged animals. The authors adeptly compare the different animal models used and provide reasons why 1 animal model is preferred to another. Biodefense: Research Methodology and Animal Models will certainly benefit scientists designing aerobiology studies or those exploring the infectious agents and toxins discussed in this book. All research laboratories focused on biodefense investigation should seriously consider adding this text to their reference library.

